# Response of the Istituto Nazionale Tumori of Milan Head & Neck Cancer Unit to the COVID-19 outbreak

**DOI:** 10.1186/s41199-020-00054-6

**Published:** 2020-05-06

**Authors:** Carlo Resteghini, Paola Maggioni, Vito Di Martino, Lisa Licitra, on behalf of Head and Neck Cancer Medical Oncology Unit, Fondazione IRCCS Istituto Nazionale dei Tumori Milan, Italy

**Affiliations:** 1 Head and Neck Cancer Medical Oncology Unit, Fondazione IRCCS Istituto Nazionale dei Tumori Milan, Milan, Italy; 2 University of Milan, Milan, Italy

In a time of uncertainties such the present days, we all look for guiding principles. When no guidelines are available, principles shape our actions. *Above all, do no harm* has been ours as we are facing the largely unknown challenge of the global COVID-19 epidemic. This mentality guided our efforts to strike the balance between reducing exposure of patients and health care providers to risk of infection on the one hand, and on the other, meeting our daily goal of providing optimal oncologic treatment in a tertiary center of national relevance.

We acted in an ever-changing context. Starting on February 21st, the first non-imported Covid-19 positive cases were recorded across Northern Italy. Between February 25th and March 4th, three different urgent decrees of the Italian Prime Minister progressively locked down the entire peninsula in an effort to contain the spread of the novel coronavirus SARS-CoV-2. In a matter of a few hours, our patients’ ability to reach our center was impaired due to logistical reasons. Public transportation was drastically reduced; those who had been in a high-incidence area such as our Institute’s region were required to enter a 2-week self-quarantine upon return to their homes.

While general hospitals across our region struggled to organize the response, which naturally focused on converting near-all activities to assist COVID-19 patients, our Institute’s mission to treat oncologic patients was preserved through measures implemented over a course of 2 weeks. A surveillance department within our center was created in order to screen oncologic patients with symptoms of infection and to contain already admitted subjects who developed the disease. Screening at the Institute’s entrance was put in place, restricting caregivers’ access to selected cases and blocking all subjects with body temperature above 37,5 °C (99,5 °F), proposing them COVID-19 tests. Hand sanitizer and personal protective equipment (PPE) such as surgical masks were also distributed at the entrance. Social distancing was ensured in the waiting rooms by restricting sitting areas.

Our unit, which is fully dedicated to medical treatment of head and neck cancer (HNC) patients (see Table 1 for activity details), adopted some additional preventive measures beginning February 24th. We report here about the organizational impact of all implemented measures on a special cancer population.

**Table 1:** Summary of 2018 Activity of the Head and Neck Cancer Medical Oncology Unit at Milan National Cancer Institute

Activity	Number
Number of admitted HNC pts	485
Number of IV systemic therapies to inpatients	374
curative	285
palliative	89
Number of outpatients visits	2582
Number of IV systemic therapies to outpatients	675
curative	107
palliative	568
Curative chemo-radiotherapy for HNC	67
Post-Operative chemo-radiotherapy for HNC	19

All patients with scheduled in- or out-patient visits were contacted the day prior to the visit to assess for fever, flu-like symptoms, and recent travel history – especially international travel, as well as for exposure to know COVID-19 cases in the previous 3 weeks. Patients with flu-like symptoms were referred to the emergency service for proper screening, while subjects with high exposure risk (e.g.: residing in high incidence areas) were rescheduled. Furthermore, to minimize the risk of exposure, long-term follow-up visits for patients treated with curative intent (ie, those patients on observation for over 2 yrs) were generally rescheduled. Subjects on watchful waiting, such as those with slowly evolving differentiated thyroid carcinomas or salivary gland cancers, were asked to send in already performed radiologic exams, skipping the clinical evaluation while still providing a feedback on disease evolution. Oral treatments such as tyrosine kinase inhibitors (TKI) and androgen-deprivation therapy (ADT) were delivered to patients’ homes, provided they executed and shared with their clinician routine tests and did not report any new toxicity. We guaranteed treatment continuity to subjects with curable disease and all of those with ongoing intravenous therapies.

Symptomatic patients with rapidly evolving oncological disease and those near the end of life represented a challenge. Whenever possible, referral to home services for symptomatic relief was undertaken. Working in coordination with the regional emergency service, septic/acute patients were directed to the most appropriate center – either a COVID-19-dedicated hospital in highly suspect cases, or to our Institute when tumor-related conditions were deemed more probable.

Of note, some patients autonomously rescheduled their appointments out of fear of being exposed to the new coronavirus while accessing to our Institute.

Maintenance of staff health throughout this epidemic is of paramount importance. Given the high COVID-19 incidence in our area, each subject should be considered a potential case. Therefore, adequate PPE were employed while visiting outpatients and frequent cleaning of offices and medical devices was implemented. While few COVID-19 cases were detected in our institutes’ patient population, a rising number of health care providers were exposed. Therefore, whenever possible remote work was adopted to reduce the diffusion risk among employees.

**Fig. 1:**
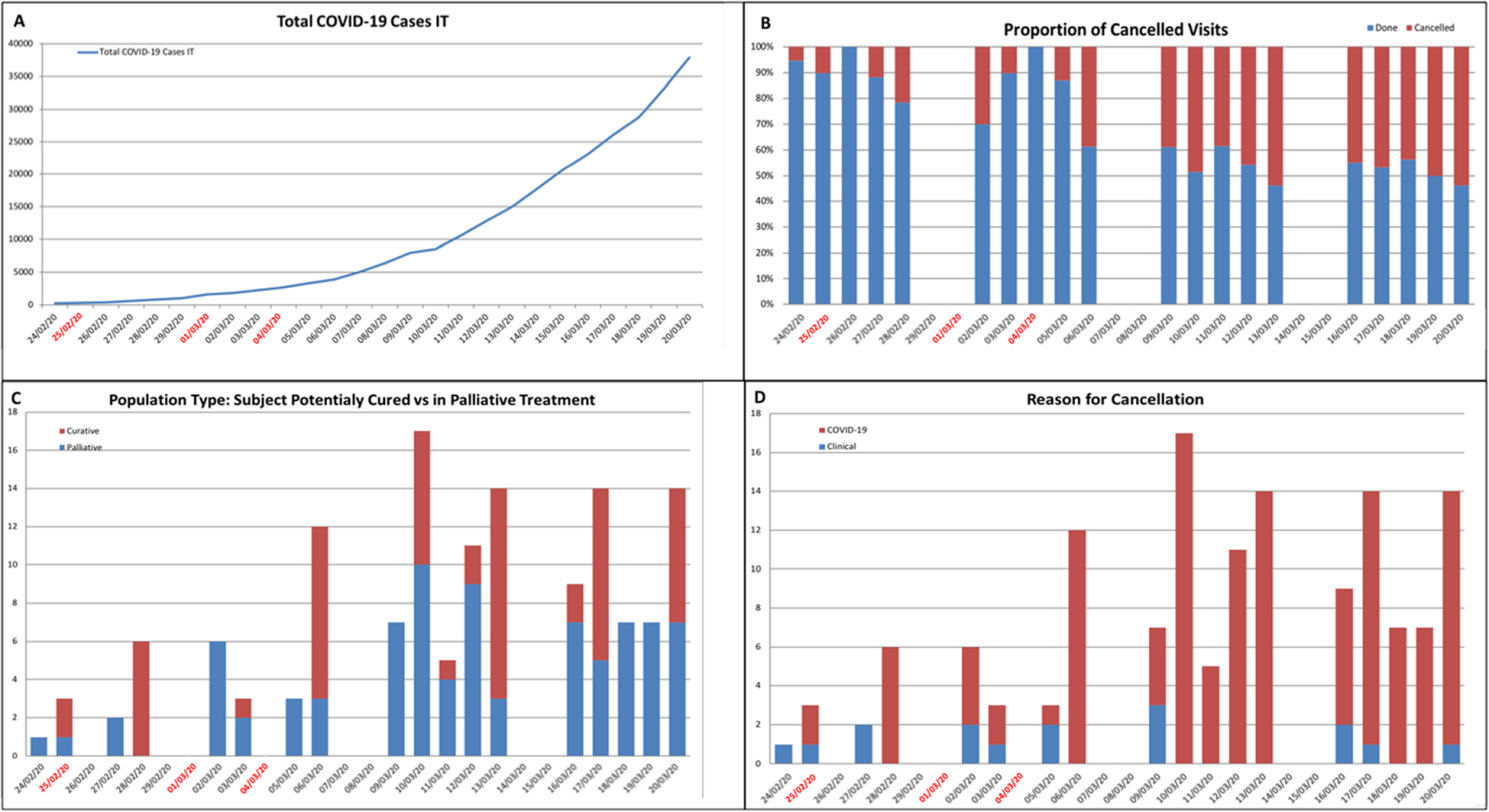
Dynamic of rescheduled patients (pts) at the Head and Neck Cancer Medical Oncology Unit at Milan National Cancer Institute in relationship with national COVID-19 cases growth and the ever more stringent lockdown policies. **a** Total confirmed positive Covid-19 cases in Italy; **b** Proportion of cancelled (red) and performed (blue) visits; **c** Characteristics of the rescheduled subjects according to treatment intent (curative in red vs palliative in blue) and **d** reason for visit cancellation (logistic causes related to Covid-19 in red, other clinical reason in blue). In the abscissa we signaled in red the date of urgent decrees of the Italian Prime Minister which progressively locked down the country

We are aware that the pandemic side-effects will be perceived much longer than the outbreak itself. Therefore, since February 24th we have tracked the dynamic of rescheduled patients in relationship with national COVID-19 case growth and the ever more stringent lockdown policies (Fig. 1a). Data in Fig. 1b shows the steep increase in rescheduled visits after travel restriction policies took place, with visit activity reduced in half in the second and third weeks of March. We differentiated the reasons for visit rescheduling between clinical causes (i.e.: neutropenia) and COVID-19-related causes (i.e.: flight cancellation, subject residing in red-zone areas). COVID-19-related causes represented the majority of rescheduling causes (Fig. 1d). A large proportion of rescheduled visits clustered on Tuesdays and Fridays when multidisciplinary follow-up of potentially cured patients takes place (Fig. 1c). These data reflect the difficulties in being true to the *do no harm* principle: sparing useless exposure to cured patients is as crucial as intercepting a potentially curable HNC recurrence. Careful case-by-case evaluation should be recommended while selecting the right candidate to skip possibly life-saving controls.

We also want to appreciate the proportion of HNC patients directly affected by COVID-19 infection. Therefore, we recently started an observational study investigating our units’ population on active treatment with access to our department from February 2020 up to the end of March; HNC patients on follow-up with access in the last 6 months will also be investigated. We will collect via telephone information regarding development of flu-like symptoms, province of residence in the last two months and exposure to known COVID-19 cases. Clinical data on disease characteristics and oncologic treatment will be collected and correlated to the investigations’ results.

Whenever uncertainties need to be faced, real-time data collection and information-sharing are pivotal in order to collectively design guidelines. Hopefully, our efforts can provide a meaningful piece in the mosaic of the health care response to this evolving pandemic.

